# Evaluation of the Effect of Resilience and General Quality of Life on Frailty in the Elderly

**DOI:** 10.1192/j.eurpsy.2024.433

**Published:** 2024-08-27

**Authors:** M. Tepetaş, A. Ay, E. Yücel, E. Kavlu, M. F. Önsüz, S. Metintaş

**Affiliations:** ^1^Public Health, Eskişehir Osmangazi University, Eskişehir, Türkiye

## Abstract

**Introduction:**

As the life expectancy at birth improved, the increase in the elderly population, one of the most vulnerable groups in society, brings about some problems. Frailty is a condition that increases the risk of progressive deterioration in physiological functioning, hypersensitivity to stress and adverse health outcomes. Frailty is quite common in older people. In frail older people, recovery from illnesses is delayed and the likelihood of sequelae is increased. If frailty is recognized early, the likelihood of disease sequelae and mortality can be reduced.

**Objectives:**

This study was conducted to determine the relationship between psychological resilience and quality of life on frailty in individuals aged 65 years and older admitted to hospital.

**Methods:**

The study group of this cross-sectional study consisted of 504 people who applied to an outpatient clinic at a university hospital. The Tilburg Frailty Scale, the Connor Davidson Psychological Resilience Scale Short Form and the EQ-5D-3L General Quality of Life Scale were used. The Kolmogorov-Simirnov test, the chi-square test, the Spearman correlation analysis and the multivariate logistic regression were used to analyse the data.

**Results:**

292 of the participants in the study group were men. Their ages ranged from 65 to 90 years, and the mean was 70.5±4.9 years. Scores on the Tilburg Frail Scale ranged from 0 to 14, and the mean was 6.3±2.7 points. In the study, 71.1% of participants were classified as frail. It was determined that there was a moderate negative correlation between the results of the Tilburg Frailty Scale and the results of the Connor Davidson Psychological Resilience Scale (r= -0.436) and the EQ-5D-3L VAS Scale (r=-0.608) and a strong positive correlation between the results of the EQ-5D-3L Index Scale (r=0.729)(for each p<0.001). According to multivariate logistic regression, people who did not exercise regularly were 2,33 times more frail than those who did, and people who had a health problem that required bed rest were 2,18 times more frail than those who did not.

**Conclusions:**

It was found that the frailty of people aged 65 and over is at a moderate level. An improvement in psychological resilience and general quality of life as well as an improvement in general health reduces frailty. It is recommended that people aged 65 and over to be physically active and to protect from situations that may require prolonged bed rest.

**Disclosure of Interest:**

None Declared

